# Ginseng and *Ganoderma lucidum* Use after Breast Cancer Diagnosis and Quality of Life: A Report from the Shanghai Breast Cancer Survival Study

**DOI:** 10.1371/journal.pone.0039343

**Published:** 2012-06-20

**Authors:** Ping-Ping Bao, Wei Lu, Yong Cui, Ying Zheng, Kai Gu, Zhi Chen, Wei Zheng, Xiao Ou Shu

**Affiliations:** 1 Shanghai Municipal Center for Disease Control and Prevention, Shanghai, People’s Republic of China; 2 Division of Epidemiology, Department of Medicine, Vanderbilt-Ingram Cancer Center, Vanderbilt University School of Medicine, Nashville, Tennessee, United States of America; Sookmyung Women's University, Republic of Korea

## Abstract

**Objective:**

To evaluate associations between quality of life (QOL) and use of ginseng and *Ganoderma lucidum* (*G. lucidum*) among breast cancer survivors.

**Methods:**

Included in this study were 4,149 women with breast cancer who participated in the Shanghai Breast Cancer Survival Study. Ginseng use was assessed at 6-, 18-, and 36-month post-diagnosis surveys; *G. lucidum* use was assessed at the 6- and 36-month surveys. QOL was evaluated at the 6- and 36-month surveys. Multiple linear regression models were used to examine associations between ginseng and *G.lucidum* use and QOL assessed at the 36-month survey, with adjustment for potential confounders and baseline QOL.

**Results:**

At 6 months post-diagnosis, 14.2% of participants reported regular use of ginseng and 58.8% reported use of *G. lucidum*. We found no significant associations between ginseng use at 6, 18, and 36 months post-diagnosis and participants’ total QOL score or individual scores for psychological, physical, or social well-being. Post-diagnosis *G. lucidum* use was positively associated with social well-being (adjusted mean difference: 1.26; 95% CI: 0.66, 1.86), but was inversely associated with physical well-being (adjusted mean difference: −1.16; 95% CI: −1.86, −0.47) with a dose-response pattern observed for cumulative number of times of use (*P* for trend <0.001 for both).

**Conclusion:**

We found no evidence that post-diagnosis ginseng use improved the QOL of breast cancer survivors. Post-diagnosis *G. lucidum* use was associated with better social well-being scores, but poorer physical well-being scores.

## Introduction

Breast cancer is the most prevalent cancer among women worldwide, and the number of breast cancer survivors continues to grow due to advances in early detection and treatment [1], [2], [3]. Being diagnosed and living with breast cancer is a stressful experience that affects multiple aspects of patients’ quality of life (QOL), including psychological, physical, and social well-being [4], [5], . Breast cancer patients often use herbal medicines in conjunction with conventional cancer treatments, to relieve cancer-related symptoms and boost their immune system, with the expectation of improved QOL [7], [8], [9]. However, whether post-diagnosis use of herbal medicines indeed improves breast cancer survivors’ QOL remains to be determined.

Ginseng and *Ganoderma lucidum* (*G. lucidum*) are two herbal remedies that have been widely used to proactively promote health, vitality, and longevity in Asia [10], [11], [12]. In recent years, ginseng use has gained popularity in Western countries. For instance, ginseng has been included in the Pharmacopoeias of Germany, Austria, and the United Kingdom [13]; in the United States, ginseng has been ranked as the fourth top-selling herbal medicine [14]. However, evidence-based information on the association between ginseng use and QOL is inconsistent and limited, particularly among patients with cancer [13], [15], [16]. *G. lucidum* is a popular medicinal mushroom. Besides being used for promoting health, it is also used for prevention or treatment of a variety of diseases, including cancer [12]. We have found that *G. lucidum* is one of the most commonly used herbs among Chinese breast cancer survivors [17]. Several *in vitro* studies have reported that *G. lucidum* has anti-cancer properties [12], [18], [19], [20]. However, to date, no clinical or epidemiological studies have described the influence of *G. lucidum* use after cancer diagnosis on clinical outcomes or survivor’s QOL.

The purpose of this study was to systematically evaluate associations of ginseng and *G. lucidum* use with the QOL of breast cancer survivors during the first 36 months after diagnosis in a large, population-based cohort study of breast cancer in Shanghai, China. This study provides a prospective view of the association between ginseng and *G. lucidum* use and QOL.

## Materials and Methods

### Ethics Statement

The study was approved by the institutional review boards of all participating institutions: the Shanghai Municipal Center for Disease Control and Prevention and Vanderbilt University. Written, informed consent was obtained from all study participants.

### Study Population

Study participants were breast cancer patients who enrolled in the Shanghai Breast Cancer Survival Study (SBCSS), a population-based, prospective study conducted in Shanghai, China. Details on the study’s design and implementation have been described previously [21], [22], [23], [24]. Briefly, through the population-based Shanghai Cancer Registry, 6,299 women aged 20–75 years with breast cancer were identified between March 2002 and April 2006; 5,042 (80%) provided written informed consent and enrolled in the study approximately 6 months (range: 3–11 months) after cancer diagnosis. The cohort was followed through in-person interviews at 18 and 36 months post-diagnosis. A total of 4,572 completed the 18-month interview and 4,149 completed the 36-month interview. Included in the present study were 4,149 participants who completed the baseline survey (at 6 months after diagnosis) and the 36-month interview with QOL information. There were no significant differences in socio-demographics between all participants enrolled (n = 5,042) and those included in the present study (n = 4,149) (data not shown).

### Data Collection

The baseline in-person interviews were conducted by trained interviewers using structured questionnaires that covered demographic characteristics, menstrual and reproductive history, dietary intake, use of complementary and alternative medicine (CAM), exercise participation, and family history of breast cancer. Height, weight, waist circumference, and hip circumference were measured according to a standard protocol. Body mass index (BMI) was calculated as weight (in kilograms) divided by height (in meters) squared. Clinical information collected included tumor-node metastasis (TNM) stage at diagnosis, estrogen receptor (ER) and progesterone receptor (PR) status, and primary cancer treatments received. In addition, inpatient medical charts were reviewed to verify diagnosis and therapy information. Follow-up interviews were conducted at 18 and 36 months after diagnosis to obtain information on disease recurrence, survival status, and QOL and to update information on active lifestyle factors, use of supplements, current health condition, and medication use. The Charlson comorbidity index was calculated based on a validated comorbidity scoring system [25] and the diagnostic codes from the International Classification of Disease, Ninth Revision, Clinical Modification [26].

For each participant, information on regular use (at least once a week for one month or longer) of supplements, including ginseng and *G. lucidum,* fish oil, shark cartilage, melatonin, lecithin, and vitamin supplements after diagnosis, duration (in months) of use, and frequency (times/month) was obtained. At the baseline survey, participants reported use during the period after breast cancer diagnosis. At subsequent surveys, participants reported supplement use since the last interview (i.e., for the preceding 12 months and 18 months). Information on types of ginseng and ginseng products, including red or white Asian ginseng, American ginseng, and ginseng products (tablets, capsules, extracts, etc.), was collected. Data on *G. lucidum* use were only collected at the 6- and 36-month surveys. In the current study, we focused on evaluation of ginseng or *G. lucidum* use and QOL, because these were the most common herbal preparations taken by breast cancer patients in our study population and they are specifically labeled as aids for cancer recovery on the market.

The average frequency of use after diagnosis (times/month) was calculated. We computed the cumulative number of times of use after diagnosis as “duration (months) of use × frequency (times/month)” summed over three surveys (for ginseng) or two surveys (for *G. lucidum*). We also derived patterns of ginseng or *G. lucidum* use according to information gathered at multiple surveys (the 6-, 18-, and 36-month post-diagnosis surveys for ginseng and the 6- and 36-month post-diagnosis surveys for *G. lucidum*) as follows: 1) “never users” refers to participants with no use reported at any survey; 2) “quitters” refers to participants who reported use at earlier survey(s), but stopped use subsequently; 3) “pick-up users” refers to participants who reported use only after the baseline or 18-month surveys; and 4) “consistent users” refers to participants who reported use of ginseng or *G. lucidum* on all relevant follow-up surveys (three for ginseng; two for *G. lucidum*).

Two previously validated instruments for assessment of QOL, the General Quality of Life Inventory-74 (GQOLI-74, used in the first set of 2,500 SBCSS participants) and the Short-Form Health Survey (SF-36, Chinese version, used in the second set of 2,542 SBCSS participants), were used to assess health-related QOL among participants at the baseline survey [24]. The current analysis includes 1,845 survivors who took the GQOLI-74, and 2,304 survivors who took the SF-36 at the baseline survey. At the 36-month interview, all survivors’ QOL was assessed using the GQOLI-74. The GQOLI-74 is based on the World Health Organization’s Quality of Life Assessment Instrument and was modified for use in Chinese populations. The GQOLI-74, described in detail in our previous studies [22], [27], includes 20 facets, a global QOL assessment, and covers the following 4 domains: physical well-being, psychological well-being, social well-being, and material well-being. Participants’ responses were converted to a score on a 0–100 scale for each domain and facet; higher scores reflected higher QOL. In the present study, the sexual functioning score was excluded from the calculation of physical well-being and total QOL for the GQOLI-74 instrument, because ∼93% of participants at the baseline survey and ∼92% at the 36-month survey reported “none or little” sexual activity during the 2–4 weeks before the interview. The SF-36 is composed of 16 questions with 36 items in eight health subscales. Each subscale and summary scale has a value ranging from 0 to 100. The validity of SF-36 has been evaluated in the Chinese population [28], [29].

### Statistical Analysis

The primary outcomes of this analysis included total QOL, four well-being domains, and all facets except for the sexual functioning score based on the QOL assessment at the 36-month survey. Differences in socio-demographic and clinical characteristics by ginseng and *G. lucidum* use at baseline were evaluated using Student’s t test for continuous variables and the χ^2^ test for categorical variables. Multiple linear regression models were used to estimate the mean differences and 95% confidence intervals (95% CI) for QOL scores across ginseng and *G. lucidum* categories. The following covariates were included in the multivariate models: age at diagnosis, educational level, income, marital status, exercise participation, tea consumption, menopausal status, menopausal symptoms, comorbidity, body mass index (BMI), vitamin supplement use, traditional Chinese medicine (TCM) use, TNM stage, type of surgery, chemotherapy, radiotherapy, tamoxifen use, ER/PR status, recurrence/metastasis, and baseline total QOL score. We found no clear evidence of collinearity (i.e., variance inflation factor >10) for the variables included in the final model.

We evaluated associations of patterns of use of ginseng or *G. lucidum*, average frequency, and cumulative number of times of ginseng/*G. lucidum* use and QOL at 36 months post-diagnosis. In addition, analyses stratified by baseline QOL, comorbidity, radiotherapy, and ER/PR status were carried out. Tests for interaction were conducted by comparing the model with both interactive terms and main effect terms with the model with only main effect terms. Tests for trend in the analyses were completed by entering the categorical variables as continuous parameters in the corresponding models.

All reported *P* values are two-sided, and the significance levels were set at *P*<0.05. All statistical analyses were performed by using SAS software, version 9.2 (SAS Institute Inc., Cary, North Carolina).

## Results

Of the 4,149 participants who completed the 36-month survey (range: 32.7–44.4 months after cancer diagnosis), 14.2%, 12.8%, and 10.9% reported ginseng use at the 6-, 18-, and 36-month surveys, respectively; and 58.8% and 36.2% reported *G. lucidum* use at the 6- and 36-month surveys, respectively.

Demographic, lifestyle, and medical characteristics of all participants and by ginseng or *G. lucidum* use at the baseline interview are presented in Table 1. The mean age at diagnosis was 53.8 years (±10.0 SD). At baseline, ginseng users, compared with non-users, were older and were more likely to take vitamins and other supplements, have a higher comorbidity index, be regular cigarette smokers, have undergone tamoxifen treatment, and report a better overall QOL at study enrollment, but were less likely to use *G. lucidum* or other TCM, be post-menopausal, or to have undergone mastectomy or chemotherapy. Compared with non-users of *G. lucidum*, *G. lucidum* users were younger and were more likely to use vitamins and TCM, have better education and higher income, be married, be post-menopausal, have menopausal symptoms, have a lower comorbidity index, regularly participate in exercise, have received chemotherapy and radiotherapy, and were less likely to be regular cigarette smokers.

**Table 1:** Ginseng and *G. lucidum* intake at the 6-month post-diagnosis survey (baseline) by demographic, lifestyle, and medical characteristics, Shanghai Breast Cancer Survival Study, 2002–2008.

Characteristics	Total (N = 4,149)	Ginseng		*G. lucidum*	
		No (N = 3,562)	Yes (N = 587)	No (N = 1,709)	Yes (N = 2,440)
Age at diagnosis (year)	53.8(10.0)	53.4 (9.9)	55.9(10.5)*	55.4(10.6)	52.6(9.4)*
TCM (%)	77.2	78.1	71.4*	72.9	80.2*
Vitamin supplement use (%)	36.8	35.4	45.5*	33.8	38.9*
Ginseng use (%)	14.2			15.6	13.1*
*G. lucidum* use (%)	58.8	59.5	54.5*		
Other supplement use (%)	6.31	5.42	11.75*	6.20	6.39
Educational level (%)					
No formal education or elementary school only	12.0	11.5	15.0	18.5	7.4
Middle or high school	72.6	73.0	69.9	70.2	74.3
College or higher	15.5	15.5	15.2	11.3	18.3*
Income (yuan/month per capita)					
< 1000	57.4	57.0	59.8	66.7	50.8
1000–1999	31.0	31.3	29.1	25.3	35.0
≥ 2000	11.6	11.7	11.1	8.0	14.2*
BMI	24.2(3.4)	24.1(3.4)	24.4(3.5)	24.4(3.5)	24.0(3.3)*
Marital status: married (%)	87.6	87.9	85.5	84.1	90.0*
Post-menopausal (%)	48.0	48.9	42.4*	42.4	51.9*
Menopausal symptoms (%)	64.6	65.0	62.0	60.9	67.2*
Charlson comorbidity index ≥1 (%)	20.6	19.9	24.9*	23.1	18.9*
Exercise participation (%)	65.7	65.7	65.4	63.6	67.1*
Regular alcohol consumption (%)	96.8	3.1	3.9	3.0	3.3
Regular cigarette smoking (%)	97.4	2.4	3.9*	3.9	1.8*
Regular tea consumption (%)	23.5	23.3	24.5	22.1	24.4
Mastectomy (%)	94.4	94.8	92.5*	94.0	94.8
Chemotherapy (%)	91.0	91.4	88.3*	86.4	94.2*
Radiotherapy (%)	30.5	31.0	27.4	26.6	33.3*
Tamoxifen use (%)	53.5	52.8	57.4*	54.5	52.8
TNM stage (%)					
0–I	38.3	38.9	34.6	36.8	39.3
IIA	33.2	32.8	36.1	33.7	33.0
IIB+	16.0	16.1	15.2	16.5	15.6
III-IV	8.0	7.9	8.5	7.8	8.1
Unknown	4.5	4.3	5.6	5.3	4.0
Total QOL Score	59.6(14.0)	59.4(14.1)	60.7(13.0)*	59.6(13.9)	59.6(14.0)

Note: Abbreviations: TCM, traditional Chinese medicine; QOL, quality of life.

Values are presented as means (standard deviation) or percentages.

* *P* value <0.05, for tests of the difference between women with and without regular ginseng/*G. lucidum* use.

We found no overall associations between ginseng use during the three time periods we assessed and total QOL score or scores for the physical, psychological, social, or material well-being domains measured at the 36-month follow-up (Table 2). Subdomain analysis showed that a lower score for daily living capability and a higher score for community services was associated with ginseng use at the baseline survey, a higher score for body/self-image was associated with ginseng use assessed at 18-month survey, and a lower score for the financial situation subdomain was associated with ginseng use assessed at the 36-month survey.

**Table 2:** Total, domain, and facet QOL scores at 36 months after cancer diagnosis in association with ginseng use status assessed at different surveys, Shanghai Breast Cancer Survival Study, 2002–2008.

QOL at 36 months after Diagnosis	6-month post-diagnosis survey (baseline)			18-month post-diagnosis survey			36-month post-diagnosis survey		
	No (N = 3,562)	Yes (N = 587)		No (N = 3,525)	Yes (N = 518)		No (N = 3,696)	Yes (N = 453)	
	Score	Mean difference a	95% CI	Score	Mean difference a	95% CI	Score	Mean difference a	95% CI
Total QOL score	63.19	−0.42	−1.07,0.23	63.13	0.01	−0.68,0.70	63.23	−0.46	−1.20,0.27
Physical well-being	65.57	−0.88	−1.79,0.02	65.53	−0.42	−1.38,0.54	65.56	−0.34	−1.35,0.68
Sleep and energy	61.34	−0.74	−2.06,0.59	61.35	−0.69	−2.10,0.71	61.36	−0.48	−1.97,1.01
Physical discomfort	66.48	−0.55	−2.01,0.91	66.62	−1.22	−2.77,0.33	66.52	−0.47	−2.11,1.17
Eating function	68.74	−1.06	−2.19,0.07	68.55	0.07	−1.13,1.27	68.67	−0.26	−1.53,1.01
Daily living capability	65.73	−1.18	−2.18, −0.18*	65.61	0.15	−0.92,1.21	65.68	−0.14	−1.26,0.99
Psychological well-being	69.27	−0.69	−1.52,0.14	69.16	0.26	−0.62,1.14	69.30	−0.58	−1.51,0.35
Psychological distress	78.06	−0.75	−1.82,0.32	77.99	0.07	−1.06,1.20	78.09	−0.85	−2.05,0.35
Negative feelings	71.20	−0.74	−1.76,0.28	71.28	−0.72	−1.80,0.35	71.19	−0.62	−1.76,0.52
Positive feelings	67.73	−1.11	−2.55,0.33	67.57	0.08	−1.45,1.61	67.84	−1.60	−3.22,0.03
Cognition functioning	65.58	−0.70	−1.85,0.45	65.41	0.72	−0.50,1.95	65.60	−0.11	−1.40,1.19
Body/Self-image	63.79	−0.20	−1.24,0.84	63.60	1.11	0.00,2.21*	63.81	0.23	−0.94,1.40
Social well-being	65.64	−0.36	−1.15,0.43	65.58	0.07	−0.77,0.91	65.69	−0.17	−1.06,0.72
Social support	65.31	−0.55	−2.06,0.96	65.25	−0.18	−1.78,1.43	65.50	−0.92	−2.61,0.78
Interpersonal relationships	74.37	−0.90	−1.95,0.14	74.30	−0.61	−1.73,0.50	74.36	−0.36	−1.54,0.82
Work and study capacity	57.80	−0.04	−1.03,0.96	57.73	0.50	−0.55,1.56	57.80	0.79	−0.33,1.91
Recreational and leisure activities	57.54	0.41	−0.63,1.44	57.57	0.20	−0.90,1.30	57.65	−0.39	−1.56,0.78
Marriage and family relationships	73.25	−0.75	−1.95,0.45	73.11	0.40	−0.88,1.67	73.22	0.00	−1.35,1.35
Material well-being	51.22	0.41	−0.59,1.41	51.27	−0.13	−1.19,0.94	51.37	−0.94	−2.06,0.19
Housing situation	68.27	0.22	−1.33,1.78	68.25	−0.25	−1.90,1.40	68.37	−0.98	−2.73,0.76
Community services	43.80	1.44	0.07,2.80*	43.95	0.12	−1.33,1.57	43.92	0.97	−0.56,2.50
Living function	50.46	−0.04	−1.82,1.74	50.53	−0.35	−2.24,1.53	50.59	−1.29	−3.29,0.71
Financial situation	42.38	0.02	−1.49,1.53	42.34	−0.02	−1.63,1.59	42.59	−2.45	−4.15, −0.75*
General QOL	58.93	−0.85	−1.91,0.22	58.75	0.64	−0.49,1.77	58.88	−0.02	−1.21,1.18

Note: Abbreviations: CI, confidence interval; QOL, quality of life.

a Obtained from multiple linear regression models adjusted for age at diagnosis, educational level, income, marital status, exercise participation, tea consumption, menopausal status, menopausal symptoms, comorbidity, body mass index (BMI), vitamin supplement use, traditional Chinese medicine (TCM) use, TNM stage, type of surgery, chemotherapy, radiotherapy, tamoxifen use, ER/PR status, recurrence/metastasis, and baseline total QOL.

* *P* value <0.05.

*G. lucidum* use at the 6- and 36-month surveys was not significantly associated with total QOL or psychological well-being at the 36-month follow-up (Table 3). However, we observed significant associations with higher scores for social well-being and material well-being and with a lower score for physical well-being. Specifically, *G. lucidum* users had higher scores for self-image, social support, interpersonal relationships, housing situation, and community services, but lower scores for sleep and energy, physical discomfort, and eating function.

**Table 3:** Total, domain and facet QOL scores at 36 months after cancer diagnosis in association with *G. lucidum* use assessed at 6 and 36 months after cancer diagnosis, Shanghai Breast Cancer Survival Study, 2002–2008.

QOL at 36 months after diagnosis	6-month post-diagnosis survey (baseline)			36-month post-diagnosis survey		
	No (N = 1,709)	Yes (N = 2,440)		No (N = 2,648)	Yes (N = 1,501)	
	Score	Mean difference a	95% CI	Score	Mean difference a	95% CI
Total QOL score	62.32	0.31	−0.17,0.78	62.82	0.08	−0.40,0.56
Physical well-being	65.33	−0.68	−1.34, −0.02*	65.77	−1.32	−1.99, −0.66*
Sleep and energy	61.40	−0.86	−1.82,0.10	61.72	−1.59	−2.57, −0.62*
Physical discomfort	66.98	−1.53	−2.59,-0.48*	67.06	−1.89	−2.96, −0.82*
Eating function	68.53	−0.49	−1.32,0.33	68.87	−1.11	−1.94, −0.28*
Daily living capability	64.44	0.16	−0.57,0.89	65.42	−0.69	−1.42,0.05
Psychological well-being	68.73	−0.10	−0.71,0.50	68.88	0.21	−0.40,0.82
Psychological distress	77.95	−0.29	−1.06,0.49	77.91	0.00	−0.78,0.79
Negative feelings	71.35	−0.71	−1.45,0.03	71.01	0.13	−0.61,0.88
Positive feelings	67.48	−0.64	−1.69,0.41	67.39	0.02	−1.04,1.08
Cognition functioning	64.12	0.36	−0.48,1.19	64.93	0.04	−0.81,0.89
Body/Self-image	62.74	0.77	0.01,1.52*	63.21	0.78	0.02,1.55*
Social well-being	63.88	1.20	0.62,1.77*	64.84	0.85	0.27,1.43*
Social support	61.84	2.57	1.48,3.67*	63.48	2.29	1.19,3.40*
Interpersonal relationships	72.41	1.75	0.99,2.51*	73.40	1.29	0.52,2.06*
Work and study capacity	56.14	0.70	−0.02,1.42	57.11	0.43	−0.30,1.17
Recreational and leisure activities	56.95	0.43	−0.32,1.18	57.44	−0.03	−0.80,0.73
Marriage and family	72.11	0.54	−0.34,1.41	72.82	0.23	−0.65,1.11
Material well-being	50.36	0.81	0.08,1.54*	50.78	0.52	−0.21,1.26
Housing situation	66.87	1.45	0.32,2.58*	67.37	1.40	0.26,2.54*
Community services	43.11	1.17	0.18,2.16*	43.82	0.13	−0.87,1.13
Living function	49.74	0.13	−1.16,1.42	49.86	0.52	−0.79,1.82
Financial situation	41.73	0.47	−0.63,1.57	42.09	0.06	−1.05,1.18
General QOL	58.25	−0.01	−0.79,0.76	58.79	−0.50	−1.29,0.28

Note: Abbreviations: CI, confidence interval; QOL, quality of life.

a Obtained from multiple linear regression models adjusted for age at diagnosis, educational level, income, marital status, exercise participation, tea consumption, menopausal status, menopausal symptoms, comorbidity, body mass index (BMI), vitamin supplement use, traditional Chinese medicine (TCM) use, TNM stage, type of surgery, chemotherapy, radiotherapy, tamoxifen use, ER/PR status, recurrence/metastasis, and baseline total QOL.

* *P* value <0.05.

We also evaluated associations of pattern of use, average frequency of use, and cumulative number of times of use of ginseng or *G. lucidum* since cancer diagnosis with QOL at the 36-month survey (Table 4). In general, there were no significant differences in total or subscale QOL scores between participants who never used ginseng regularly and those who ever used ginseng regularly after cancer diagnosis. We found that ginseng users in the “consistent” group, compared with never-users, had a lower score for physical well-being, which reached borderline statistical significance (adjusted mean difference: −0.71; 95% CI: −2.49, 0.07). A borderline significant association between higher frequency of ginseng use (>11.6 times/month) and low physical well-being was also observed (adjusted mean difference: −0.92 (95% CI: −1.90, 0.05).

**Table 4:** Adjusted mean differences^a^ in total QOL and domain QOL scores assessed at 36 months after cancer diagnosis by pattern and cumulative level of ginseng and *G. lucidum* use post-diagnosis, Shanghai Breast Cancer Survival Study, 2002–2008.

Status of use	No.	Total QOL score		Physical well-being		Psychological well-being		Social well-being	
		Mean difference	95% CI	Mean difference	95% CI	Mean difference	95% CI	Mean difference	95% CI
**Ginseng**									
Ever use after diagnosis									
Never users	3132	Ref	–	Ref	–	Ref	–	Ref	–
Ever users	1017	−0.03	−0.56,0.51	−0.25	−0.99,0.50	−0.13	−0.81,0.55	0.09	−0.56,0.74
Quitters	399	0.08	−0.69,0.86	-0.61	−1.68,0.47	−0.04	−1.02,0.94	−0.01	−0.95,0.93
Pick-up users	265	0.22	−0.72,1.16	0.27	−1.04,0.57	0.39	−0.80,1.59	0.40	−0.74,1.54
Consistent users	136	−0.57	−1.85,0.71	−0.71	−2.49,0.07	−0.61	−2.24,1.02	−0.32	−1.88,1.23
Other	217	−0.19	−1.22,0.84	0.10	−1.33,0.53	−0.65	−1.95,0.66	0.17	−1.08,1.41
Average frequency of use after diagnosis									
Never	3132	Ref	–	Ref	–	Ref	–	Ref	–
≤11.6 times/m	505	0.02	−0.68,0.73	0.25	−0.73,1.22	−0.45	−1.34,0.45	0.29	−0.56,1.14
>11.6 times/m	504	−0.16	−0.87,0.54	−0.92	−1.90,0.05	0.12	−0.77,1.02	−0.19	−1.04,0.67
*P* trend			0.70		0.14		0.93		0.87
Cumulative number of times of use after diagnosis									
Never	3132	Ref	–	Ref	–	Ref	–	Ref	–
≤156 times	508	0.33	−0.37,1.03	0.31	−0.66,1.28	−0.05	−0.94,0.84	0.54	−0.31,1.39
>156 times	508	−0.37	−1.08,0.33	−0.80	−1.78,0.18	−0.20	−1.10,0.70	−0.35	−1.21,0.51
*P* trend			0.54		0.22		0.68		0.77
***G. lucidum***									
Ever use after diagnosis									
Never users	1367	Ref	–	Ref	–	Ref	–	Ref	–
Ever users	2782	0.23	−0.26,0.73	−1.16	−1.86,−0.47	0.05	−0.59,0.68	1.26	0.66,1.86
Quitters	1281	0.26	−0.32,0.84	−0.63	−1.43,0.17	−0.08	−0.82,0.65	1.10	0.40,1.80
Pick-up users	342	−0.10	−0.99,0.78	−1.82	−3.04,−0.59	0.44	−0.68,1.57	0.70	−0.37,1.77
Consistent users	1159	0.31	−0.29,0.92	−1.59	−2.43,−0.76	0.08	−0.69,0.85	1.64	0.91,2.37
Average frequency of use after diagnosis									
Never	1367	Ref	–	Ref	–	Ref	–	Ref	–
<31.8 times/m	1388	−0.07	−0.64,0.49	−1.46	−2.25,−0.68	−0.28	−1.00,0.44	0.91	0.22,1.59
≥31.8 times/m	1388	0.56	−0.01,1.13	−0.86	−1.66,−0.06	0.40	-0.33,1.13	1.64	0.95,2.34
*P* trend			0.05		0.04		0.27		<0.0001
Cumulative number of times of use after diagnosis									
Never	1367	Ref	–	Ref	–	Ref	–	Ref	–
≤360 times	1457	0.13	−0.43,0.68	−0.91	−1.68,−0.14	−0.08	−0.79,0.63	1.04	0.36,1.71
>360 times	1324	0.35	−0.23,0.94	−1.49	−2.30, −0.68	0.20	−0.54,0.94	1.52	0.81,2.23
*P* trend			0.24		0.0003		0.60		<0.0001

Note: Abbreviations: CI, confidence interval; QOL, quality of life.

a Obtained from multiple linear regression models adjusted for age at diagnosis, educational level, income, marital status, exercise participation, tea consumption, menopausal status, menopausal symptoms, comorbidity, body mass index (BMI), vitamin supplement use, traditional Chinese medicine (TCM) use, TNM stage, type of surgery, chemotherapy, radiotherapy, tamoxifen use, ER/PR status, recurrence/metastasis, and baseline total QOL. Participants who never reported having regularly used ginseng or *G. lucidum* on all three surveys served as the reference.

Higher frequency (≥31.8 times/month) of *G. lucidum* use after diagnosis was related to higher total QOL scores (*P* for trend  = 0.05) and higher social well-being scores (*P* for trend <0.0001). A higher cumulative number of times (>360 times) of *G. lucidum* use after diagnosis was positively associated with social well-being (*P* for trend <0.0001; adjusted mean difference: 1.52; 95% CI: 0.81, 2.23) and inversely associated with physical well-being (*P* for trend  = 0.0003; adjusted mean difference: −1.49; 95% CI: −2.30, −0.68). These associations were predominantly observed among “pick-up” users and “consistent” users (Table 4).

Further analyses stratified by baseline QOL showed that the associations between regular ginseng/*G. lucidum* use and QOL at the 36-month follow-up were not modified by baseline QOL total score (interaction term test: all *P* >0.05) (Table 5). Similarly, we found no interaction of comorbidity, radiotherapy, or ER/PR status with regular use of ginseng or *G. lucidum* among the study participants (data not shown).

**Table 5:** Associations of ginseng and *G. lucidum* use with total QOL and domain QOL scores assessed at 36 months after cancer diagnosis stratified by baseline total QOL score, Shanghai Breast Cancer Survival Study, China, 2002–2008.

Baseline QOL score	Ginseng					*G. lucidum*				
	Score < median (N = 2,075)		Score ≥ median (N = 2,074)		*P* for interaction	Score < median (N = 2,075)		Score ≥ median (N = 2,074)		*P* for interaction
	Mean difference a	95% CI	Mean difference a	95% CI		Mean difference a	95% CI	Mean difference a	95% CI	
Total QOL score	0.06	−0.74,0.85	0.06	−0.69,0.81	0.88	0.40	−0.34,1.14	−0.15	−0.86,0.55	0.17
Physical well-being	0.05	−1.03,1.14	−0.33	−1.38,0.72	0.82	−1.27	−2.28,0.26	−1.39	−2.37,0.41	0.62
Psychosocial well-being	−0.19	−1.24,0.85	0.07	−0.85,0.99	0.71	0.29	−0.68,1.26	−0.45	−1.31,0.42	0.25
Social well-being	0.34	−0.59,1.27	0.05	−0.88,0.98	0.78	1.57	0.70,2.43	0.80	−0.08,1.67	0.08

Note: Abbreviations: CI, confidence interval; QOL, quality of life.

a Obtained from multiple linear regression models adjusted for age at diagnosis, educational level, income, marital status, exercise participation, tea consumption, menopausal status, menopausal symptoms, comorbidity, body mass index (BMI), vitamin supplement use, traditional Chinese medicine (TCM) use, TNM stage, type of surgery, chemotherapy, radiotherapy, tamoxifen use , ER/PR status, and recurrence/metastasis. Participants who never reported having regularly used ginseng or *G. lucidum* on all three surveys served as the reference.

## Discussion

This is the first comprehensive study to evaluate the effects of regular use of ginseng and *G. lucidum* as a complementary therapy at different time points after cancer diagnosis on breast cancer survivor’s QOL in a large, population-based cohort study. We found no evidence that ginseng use post-diagnosis was associated with improved QOL at the 36-month follow-up. Survivors who used *G. lucidum* regularly after cancer diagnosis reported a higher score for social well-being, but a lower score for physical well-being, compared with non-users. These differences were more apparent for higher frequency and cumulative number of times of use during the entire follow-up period.

Ginseng has been used to maintain natural energy, increase physical and psychomotor performance, and improve mood and general health [13]. There are several types of ginseng, including Asian ginseng (*Panax ginseng*) and American ginseng (*P. quinquefolius*); the major active constituents are ginsenosides. Over 80% of our study population used American ginseng. *In vitro* experiments and *in vivo* animal studies have reported that ginsenosides have a variety of beneficial effects, including immunodulatory, anti-stress, anti-fatigue, and anti-carcinogenic effects [30], [31], [32]. However, in human studies, findings on the effects of ginseng on health-related QOL are mixed [13], [15], [16]. A systematic review by Vogler et al. summarized several randomized clinical trials and found contradictory results for ginseng ability to improve physical performance and immune function [16]. Coleman et al. reviewed 9 clinical trials and concluded that improvement in overall health-related QOL cannot be attributed to *P. ginseng*, although various facets of QOL had improved [13].

There is a paucity of data from epidemiological studies regarding the effects of ginseng use on QOL among cancer patients. Our previous studies have shown that ginseng use, particularly current use at the fourth year post-diagnosis, was positively associated with QOL scores, with the strongest effects in the psychological and social well-being domains [15]. In the present study, however, we found no significant positive associations between post-diagnosis ginseng use and survivors’ QOL at the 36-month follow-up. There are several potential reasons for this discrepancy. First, the design of previous studies differed from the present study, which is a prospective survival study specifically designed to investigate post-diagnostic lifestyle and CAM use and collected relevant information at multiple, pre-determined time points (6, 18, and 36 months after cancer diagnosis). Our previous study was an ad hoc follow-up of breast cancer patients enrolled in a case-control study, for which exposure data were collected at one time point after cancer diagnosis (approximately 48 months). Second, the prevalence of ginseng use among breast cancer survivors was substantially lower in the current study compared with the previous study, which may have obscured the association between ginseng use and breast cancer survivors’ QOL. In the previous study, 62.8% of women with breast cancer reported post-diagnosis ginseng use, and 30.6% were current users. In the present study, 14.2% of participants were ginseng users at baseline and 10.9% were ginseng users at the 36-month follow-up. In addition, the timing of the QOL assessment also differed between our two studies. In our earlier study, QOL was assessed only once at the fourth year post-diagnosis, while in the present study, QOL was assessed at 6 and 36 months post-diagnosis and the association of QOL at the 36-month survey with ginseng use was adjusted for the baseline QOL score.

*G. lucidum* was the most popular herbal remedy used by our study participants [17]. The prevalence of use among women with breast cancer increased during the decade prior to the current study’s recruitment period from 18.9% among breast cancer cases enrolled in our case-control study (1996–1998) [33] to 58.4% (2002–2006) [17]. In the present study, 58% of participants reported *G. lucidum* use at the 36-month survey. To date, over one hundred species of oxygenated triterpenes have been isolated from *G. lucidum*. *G. lucidum* has been reported to have many biological activities, such as histamine release-inhibitory action, immunomodulatory activity, antitumor cytokines acting on inhibition of leukemic-cell growth, and differentiation inducing activity [10]. *In vitro* experiments have shown that *G. lucidum* can inhibit proliferation, invasive behavior, and growth of tumor cells, and induce tumor cell apoptosis [12], [18], [19], [20], [34], [35]. However, no study has reported on the effects of *G. lucidum* use on clinical outcomes and/or QOL among breast cancer survivors. In the present study, we found that *G. lucidum* use after breast cancer diagnosis was associated with a higher score for social well-being, but a lower score for physical well-being. As a result, the overall QOL score was not significantly associated with *G. lucidum* use. The underlying mechanisms for these associations are unclear. The improvement of social well-being may reflect better financial and social support, while the reduced physical well-being score may suggest that either *G. lucidum* use negatively influenced patients’ physical well-being or patients with low physical well-being were more likely to seek *G. lucidum* as complementary treatment. Given the popularity of *G. lucidum* use among women with breast cancer, more studies should be conducted to clarify its effects on breast cancer survivors’ QOL.

Our study has several strengths. It is the first population-based, longitudinal, prospective study with a large enough sample size to investigate relationships between the use of ginseng and *G. lucidum* after diagnosis and QOL among breast cancer survivors. Multiple assessments improved the quality of both the exposure and outcome information. Selection bias was largely minimized due to the high response and follow-up rates. The detailed data on socio-demographic, lifestyle, and known clinical prognostic factors that we collected allowed a detailed adjustment for potential confounding factors. However, several limitations of our study should also be noted. First, information about pre-diagnosis use of ginseng and *G. lucidum* was not available; thus, we were unable to evaluate the influence of pre-diagnosis ginseng and *G. lucidum* use on QOL. Second, the brand of ginseng and *G. lucidum* products and specific dosages used may affect their effectiveness, but our study did not collect this information. Third, although sexual functioning is an important component of QOL, we were not able to evaluate its association with ginseng and *G. lucidum* use, because of the very small number of participants who reported being sexually active, which is likely due to the fact that the subject of “sexual functioning” is considered to be very private in Eastern cultures.

In summary, our study found no evidence that ginseng use after breast cancer diagnosis improved survivors’ QOL during the first three years post-diagnosis. *G. lucidum* use after cancer diagnosis was associated with better social well-being, but poorer physical well-being. Further studies are needed to investigate the benefits and safety of ginseng and *G. lucidum* use among longer-term cancer survivors.
